# Remotely Monitoring COVID-19 Patient Health Condition Using Metaheuristics Convolute Networks from IoT-Based Wearable Device Health Data

**DOI:** 10.3390/s22031205

**Published:** 2022-02-05

**Authors:** Mustafa Musa Jaber, Thamer Alameri, Mohammed Hasan Ali, Adi Alsyouf, Mohammad Al-Bsheish, Badr K. Aldhmadi, Sarah Yahya Ali, Sura Khalil Abd, Saif Mohammed Ali, Waleed Albaker, Mu’taman Jarrar

**Affiliations:** 1 Department of Computer Science, Dijlah University College, Baghdad 10022, Iraq; sarah.yahia@duc.edu.iq (S.Y.A.); Sura.khalil@duc.edu.iq (S.K.A.); saif.alameri@duc.edu.iq (S.M.A.); 2 Department of Medical Instruments Engineering Techniques, Al-Farahidi University, Baghdad 10022, Iraq; 3 Police Academy, Baghdad 10022, Iraq; alamery.thamer@outlook.com; 4 Computer Techniques Engineering Department, Faculty of Information Technology, Imam Ja’afar Al-Sadiq University, Nasiriyah 64001, Iraq; mohammed.hasan@uokufa.edu.iq; 5 College of Computer Science and Mathematics, University of Kufa, Najaf 540011, Iraq; 6 Department of Managing Health Services and Hospitals, Faculty of Business Rabigh, College of Business (COB), King Abdulaziz University, Jeddah 21991, Saudi Arabia; oal@kau.edu.sa; 7 Healthcare Administration Department, Batterjee Medical College, Jeddah 21442, Saudi Arabia; mohammed.ghandour@bmc.edu.sa; 8 Department of Health Management, College of Public Health and Health Informatics, University of Ha’il, Ha’il 81451, Saudi Arabia; b.aldhmadi@uoh.edu.sa; 9 Department of Computer Science, Al-Turath University College, Baghdad 10021, Iraq; 10 Department of Internal Medicine, College of Medicine, Imam Abdulrahman Bin Faisal University, Dammam 34212, Saudi Arabia; wialbakr@iau.edu.sa; 11 Medical Education Department, King Fahd Hospital of the University, Al-Khobar 34445, Saudi Arabia; mkjarrar@iau.edu.sa; 12 Vice Deanship for Quality and Development, College of Medicine, Imam Abdulrahman Bin Faisal University, Dammam 34212, Saudi Arabia

**Keywords:** COVID-19, cloud computing, deep learning, healthcare data, IoT sensors, wearable sensors

## Abstract

Today, COVID-19-patient health monitoring and management are major public health challenges for technologies. This research monitored COVID-19 patients by using the Internet of Things. IoT-based collected real-time GPS helps alert the patient automatically to reduce risk factors. Wearable IoT devices are attached to the human body, interconnected with edge nodes, to investigate data for making health-condition decisions. This system uses the wearable IoT sensor, cloud, and web layers to explore the patient’s health condition remotely. Every layer has specific functionality in the COVID-19 symptoms’ monitoring process. The first layer collects the patient health information, which is transferred to the second layer that stores that data in the cloud. The network examines health data and alerts the patients, thus helping users take immediate actions. Finally, the web layer notifies family members to take appropriate steps. This optimized deep-learning model allows for the management and monitoring for further analysis.

## 1. Introduction

COVID-1 is a critical and challenging disease to identify in its earliest stages [1,2,3]. One solution is to minimize its effects by taking mitigating steps to reduce the disease’s spread with the help of the Potential Infected Patient (PIP) monitoring process [4]. PIP monitoring is achieved by using the Internet of Things (IoT) [5], which monitors a patient’s activity via a wearable device. IoT devices collect, analyze, monitor, and manage disease symptoms remotely. As a result, the time is ripe to implement IoT solutions that could improve identifying those with COVID-19 and contact trace (identifying people exposed to infected people). IoT systems can track medication prescriptions, monitor patients remotely, and send and receive medical information via wearables. Thus, practitioners can more readily examine, diagnose, and treat patients by using IoT-based telemedicine technology without physical engagement. An IoT platform can be used to control drones monitoring crowds, make public announcements, screen crowds, spray disinfectants, and convey medical supplies and other essentials with minimum human involvement.

**Figure 1:**
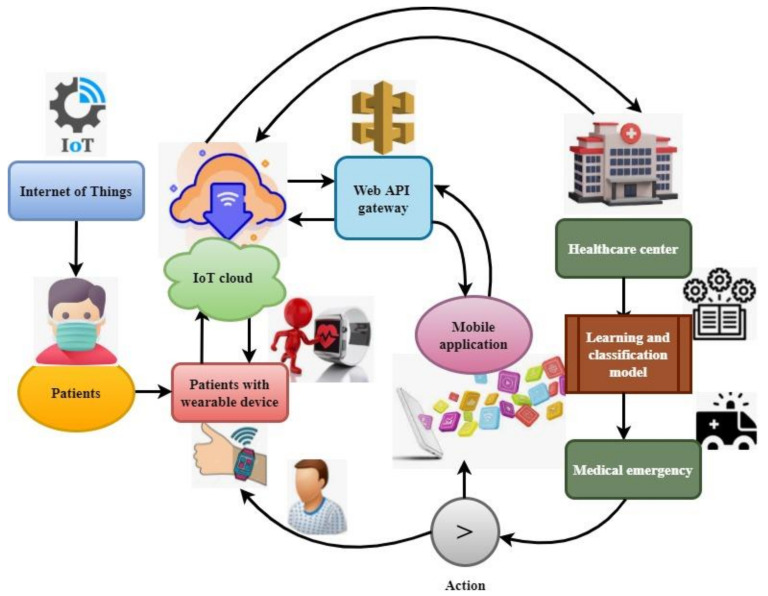
IoT with cloud involvement for the COVID-19 situation.

Wearable technologies monitor COVID-19 symptoms using a sensor device that records the patient’s temperature, oxygen saturation, heart rate, and other respiratory information. Indeed, some healthcare systems widely utilize IoT wearable devices to identify COVID-19 in the current pandemic [6,7]. The collected health details are stored in the cloud via the Internet, and clinicians can investigate the information. Figure 1 illustrates the IoT with cloud-based COVID-19-patient health monitoring. Wearable devices continuously record a patient’s physical activities and collect data that are then transferred to a healthcare center [8,9]. The gathered details are investigated using the learning and classification model to identify abnormal activities.

Several researchers have used the deep-learning model [10,11,12] because it can examine high-dimensional sensor data. The integration deep-learning model in the IoT reliably impacts a healthcare center. The IoT devices play a critical role in the COVID-19-patient monitoring process because COVID-19 has various stages. The first stage is asymptomatic [13], in which patients do not display any symptoms [14]. In the second stage, patients have a cough, cold, and fever. In the third stage, the virus spreads to other people. Last, patients recover from the disease in the fourth stage. The basic symptoms should be monitored to reduce COVID-19 impacts and help reduce the virus’ spread. Novel coronavirus symptoms [15,16], such as SpO2 (oxygen saturation), body temperature, and pulse rate, should be monitored to determine the symptoms.

These health parameters are monitored with the help of the Internet of Medical Things (IoMT) [17], and wearable devices monitor a patient’s health. According to recent research, 162 million devices are currently utilized in the medical field to capture patient health conditions. During the data-collection process, edge and fog computing are incorporated in IoT sensor devices to ensure high availability, low latency, and patient location. A deep-learning model processes details to recognize the vital signs of patients. Authorities can use the information to restrict the area in which a person travels to avoid spreading the virus [18]. Distraction, signal or battery failure, and reliance on the US Department of Defense, Privacy Issues, and Crime Commercial Exploitation are some issues that might arise when using a GPS device. Additionally, GPS, designed for outdoor use, cannot be used indoors because GPS signal attenuation in interior environments reduces precision.

Wearable sensors measure various metrics, such as motion activity, respiration rate, heart rate, body temperature, stress, cough symptoms, and oxygen saturation level. A person can communicate with a computer system through its user interface characteristics. Anything in this category can be a screen or page. Examples of user interfaces include desktop software and programs and mobile apps. These physiological measurements are stored in the cloud environment that clinicians request for clinical analysis. A system that effectively monitors patient health and COVID-19-disease-related symptoms [19] should be recognized immediately to reduce the spread of the virus.

This paper introduces an effective API-layer-based data collection and deep-learning model to aid this effort. The system also focuses on the timely escalation to track patient health. A successful monitoring process minimizes the mortality rate and improves treatment procedures. In this context, the paper discusses the following.

- IoT-wearable-devices-based COVID-19-patient health-monitoring process on minimizing the virus spread;
- The utilization of the optimized deep-learning model to maximize the disease-classification process with minimum deviations; and
- The design and implementation of a 3D model with sensors and a prototype for effectively conveying a patient’s details.

The remaining organization of the paper is the following: Section 2 discusses various researchers’ opinions regarding the COVID-19 remote patient health-monitoring process. Section 3 discusses the working process of an API-based implemented deep-learning model to identify the COVID-19 patients. Section 4 discusses the system’s effectiveness, and Section 5 concludes the study.

## 2. Related Works

This section analyzes the various opinions on the remote patient-monitoring process that uses wearable sensor devices. El-Rashidy et al. [20] recommend an end-to-end deep-learning model (ETEDL) to monitor and detect COVID-19 disease. This system integrates the fog, cloud, wireless body network, and clinical decision-making concepts. The framework uses the patient, cloud, and hospital layers to investigate the patient health conditions. The integrated sensor device and mobile app can track the patient’s health condition with up to 97.5% accuracy. The collected details are processed using a convoluted deep-learning model, which recognizes abnormal activities with up to 98.85% specificity.

Rahman et al. [21] introduced B5G and an explainable deep-learning model (BG5D EDL) to predict COVID-19 and improve healthcare. The system intends to create an effective deep-learning architecture to support data privacy, low latency, and scalability while monitoring COVID-19 patients. The B5G framework can resolve extensive training data and ethical-approval-related issues. This process uses the three-phase reconciliation global DL framework to monitor the patient’s health condition effectively.

Tan et al. [22] developed 5G-enabled wearable devices and a deep-learning approach to monitoring a patient’s health condition. This system addresses real, timely, and efficiency-related issues while monitoring COVID-19 patients. The 5G device gathers patient electrocardiogram information processed by convolution and extended short-term network. The approach investigates the data characteristics and identifies the cardiovascular health condition with 99.29% accuracy.

Rathee et al. [23] created a COVID-19-patient monitoring system by integrating the Internet of Things (IoT) and Artificial Neural Networks (ANNs). This system recognizes Covid-19 patients by maintaining the system’s suitability and applicability. The created system classifies COVID-19 patients into susceptible (ST), exposed (EP), uninfected (UI), and infected people (IN). This system recognizes the COVID-19 patients accurately because of the effective Bayesian and backpropagation-algorithm-based training process.

Otoom et al. [24] developed an IoT-framework-based Covid-19 patient detection system to minimize the mortality rate. The wearable sensors gather patient health information stored in the cloud infrastructure. The shared data that machine-learning techniques, such as KNN, SVM, neural network, and naïve Bayes approach, examined recognize the disease up to 90%. These classification results help to improve the treatment procedure and reduce the mortality rate.

Ucar et al. [25] recommend an optimized deep Bayes squeeze network (ODBSN) to recognize the COVID-19 disease from radiographic images. The developed system aims to reduce the false-recognition rate and minimize the computation time. The Bayesian optimization algorithm is utilized during the classification process to tune the neural network for improving the recognition rate. The effective utilization of the tuning parameter improves the overall recognition accuracy.

Mukherjee et al. [26] applied an enhanced K-Nearest Neighboring classifier on IoT-based collected data to recognize the COVID-19 disease. This process uses the 7-benchmark dataset information from various countries, such as Mexico and Brazil. The details were examined using Ant Colony optimization techniques to select optimal features. These features are classified by using the KNN approach that recognizes COVID-19 patients with maximum accuracy.

Mahmud et al. [27] recommended using CovXNet (multi-dilation convolutional neural network) and transferable multi-receptive feature optimization techniques to recognize the COVID-19 disease from X-ray images. The system utilizes a more extensive database that consists of non-COVID pneumonia X-ray images. The collected images are examined by using a deep-learning network with dilated convolutions. The effective utilization of learning functions recognizes the COVID-19 disease with 97.4% accuracy.

Fan Yang et al. [28] defined one method for measuring vital signs without the use of a face mask, as was proposed in this research that uses latent heat and an RGB camcorder to measure a person’s temperature of the body, heartbeat, and breathing rates (BRs). There were three areas of interest (AOIs) found by using facial landmarks and the convolution neural network (CNN) face detector. A group of ten healthy individuals from a wide range of ethnic backgrounds and skin tones participated in a series of experiments.

Irfan Ullah Khan et al. [29] noted that, as the coronavirus pandemic (COVID-19) spreads around the world, it poses a serious threat to millions of people. Precise and appropriate treatment of COVID-19 is critical to halting its spread and reducing the risk of infection. The result shows that the proposed method automated the diagnosis of multiple diseases using machine learning (ML) for effective diagnosis. Based on CXR images and patient clinical data, a deep-learning model was proposed in the current study for the computer-aided diagnosis of COVID-19. This research aims to determine the impact of using CXR data in conjunction with clinical patient data to automate the COVID-19 diagnosis.

As observed from Table 1, the COVID-19 disease is recognized from radiographic images and wearable sensor information according to various researchers’ opinions. The IoT-device-collected details are more helpful in handling the patient remotely. Therefore, IoT wearable sensor devices capture the patient health information in this work. These details are processed using various machine-learning techniques to improve the recognition rate. Those methods fail to achieve high accuracy with minimum time. Hence, effective meta-heuristics convolute networks are utilized to predict a patient’s health condition and alert the relatives concerning COVID-19 status.

## 3. Materials and Methods

### 3.1. Proposed System Design

As discussed earlier, the remote health-monitoring system was designed by utilizing the Internet of Things (IoT) because it is convenient, simple, and makes it easy to access patient health information. COVID-19 sufferers could benefit significantly from IoT-based health-monitoring devices if the epidemic continues. Critical-care measurements, such as body temperature, heart rate, and oxygen saturation, can be used in real time by an IoT-based system proposed in this study. Using a liquid crystal display (LCD) connected to a mobile application makes it possible to see temperature, heart rate, and oxygen saturation levels in real time. The healthcare system uses various IoT components, such as a microcontroller, actuators, sensors, and cloud permitted systems, that help gather the patients’ health information at home rather than having them visit the hospital.

The IoT-health monitoring process investigates physiological metrics and COVID-19 symptoms transmitted to the health center via the Application Peripheral Interface (API). The API is treated as the database used to measure the disease infection level. In addition to this, the IoT sensor computes the geographical details, which help to alert the relatives when the self-quarantine people are affected by COVID-19 symptoms. The developed system has three layers: IoT, cloud, and mobile layer. Each layer has a specific function that utilizes recordings and monitors COVID-19 patients successfully. The main intention of the health monitoring framework is to alert the patient and healthcare authorities to recognise the infected people in different situations and environments.

**Figure 2:**
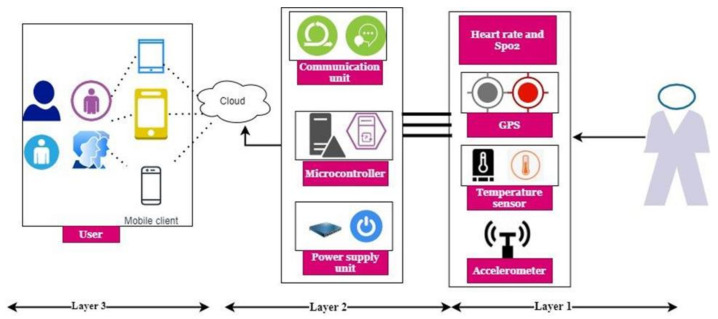
Three-layer design of COVID-19-patient health-monitoring framework.

The gathered signal information is processed by using Mel-frequency Cepstral Coefficients (MFCC) feature extraction method [30]. The MFCC based derived features are input to the neural network used to recognise the patient’s health condition. Here 24 cepstral coefficients and 0.02 frame length are utilized to extract the features. Here, the database is utilized to save the collected healthcare information and get the details for further medical analysis. According to the discussion, the three-layered IoT-designed remote health monitoring system is illustrated in Figure 2.

#### 3.1.1. Layer 1: IoT Layer

The first layer is the wearable IoT, which is accountable for collecting patient information. The details are in two types: GPS-sensor-based data and health-related information. The GPS sensor gathers location-related information to alert the patient and relatives when they are affected by COVID-19 symptoms. The other wearable sensors collect the patient’s oxygen saturation (SpO2), heart rate, temperature, and cough details. The layer has a microcontroller that recites the data from the sensor stored in the cloud via the application design. The GPS sensor gathers patients’ location details and is saved in the cloud for further medical actions.

#### 3.1.2. Layer 2: Cloud Layer

The cloud layer accepts the data from the microcontroller stored in the cloud by establishing basic security measures. Here, Cloudflare is used to develop the security of the data via Internet connections. The successive establishment of security factors maintains the healthcare application’s data reliability, scalability, and security. The collected data include the patient contacts, location, and emergency contacts stored in the Cloudflare network. These data are transferred to the authorized users via the API endpoints. Once COVID-19 affects a patient, he or she is informed about taking proper actions via SMS and email.

#### 3.1.3. Layer 3: Web Front Layer

The web front layer receives the data from the cloud system by maintaining credibility and data ownership. An ankle bracelet gathers information, such as SpO2 saturation details, temperature, heart rate, and cough information. Then latitude- and longitude-related information is gathered using a GPS sensor to identify the patient’s location. Once the data are collected, COVID-19 symptoms are examined using the neural model to investigate the patient health status. Enabling the IoT is a complex process involving many embedded systems. These include microprocessors (CPUs), sensors, and communication gear. The IoT ecosystem consists of web-enabled intelligent devices. Depending on the device and the IoT network itself, an IoT gateway or other edge device links IoT sensors to the cloud. Data are then either provided or analyzed locally. The collected information then can be used to alert a patient to take emergency actions.

### 3.2. Data Processing and Selection

Initially, the primary physiological sensor is utilized to collect the body temperature, pulse rate, and oxygen saturation because these values help identify the initial symptoms of the COVID virus infection. Here, the body-temperature threshold value is 100 bpm or 38 °C, and the oxygen saturation value is 92 to 96%. Once the patient’s health condition meets these threshold values, patients are treated as virus-infected people. According to various clinical studies, if a patient has a fever above 90% and 75% of cough affected, then that person is infected mainly by COVID-19. Therefore, the patient should be monitored for his or her cough and temperature to avoid the virus infection. The cough should be examined compared to temperature because cough varies as short, long, and dry. Hence, the trained neural model is needed to predict the patient’s health condition. The gathered signal information is processed by using Mel-Frequency Cepstral Coefficients (MFCC) feature extraction method. The MFCC-based derived features are inputted into the neural network used to recognize the patient’s health condition. Here, 24 Cepstral coefficients with a 0.02 frame length are utilized to extract the features. During the feature-extraction process, 0.02 successive frames are selected, with 32 filter banks utilized to examine the audio signal. Along with this, 256 FFT points, 101 sliding widow size, 300 Hz MEL filter lowest band edge, 800 Hz MEL highest band edge, 0.98 pre-emphasizing coefficients, 1 shift, and 800 Hz sampling frequency/2 are utilized to derive the variation of the characteristics in the patient audio signal. The defined MFCC characteristics and properties effectively extract the cough-level variation. Color channels correlate to the depth of the picture’s three-dimensional (3D) matrix representation) (RGB). Using 3D is required by design for convolution filters because they cover all the depth of their input and have a specified height and breadth of 5 × 5. Two 3 × 3 convolutions placed side by side produce the same receptive field as one 5 × 5 convolution.

(1) $$\mathrm{Peak}\;\mathrm{amplitude}=\;\left(\mathrm{amplitude}\;\mathrm{in}\;\mathrm{stochastic}\;\mathrm{period}\right)$$

(2) $$\mathrm{Harmonic}\;\mathrm{distortion}=\frac{\mathrm{Harmonic}\;\mathrm{components}}{\left(\mathrm{power}\;\mathrm{of}\;\mathrm{fundamental}\;\mathrm{frequency}\right)}$$

(3) $$\mathrm{Heart}\;\mathrm{rate}=\frac{60}{\left(\mathrm{RR}\;\mathrm{Interval}\right)}$$

(4) $$Zero\;crossing\;rate\;\left(ZCR\right)\;\mathrm{ZCR}=\mathrm{signal}\;\mathrm{sign}\;\mathrm{changes}\;\mathrm{from}\;\mathrm{positive}\;\mathrm{to}\;\mathrm{negative}$$

(5) $$Entropy=\sum_{\left(i,j=0\right)}^{\left(n-1\right)}-\ln\left(P_{ij}\right)P_{ij}\;$$

(6) $$Energy=\sum_{\left(ij=0\right)}^{\left(n-1\right)}\ln{\left(P_{ij}\right)}^{2}$$

(7) $$Standard\;Deviation\;\left(SD\right)=\frac{1}{N}\sqrt{\sum_{i=2}^{N}ln{\left(RR_{i}-RR_{i-1}-\mu \right)}^{2}}$$

The equations mentioned above, from (1) to (7), are utilized to derive the statistical features that are more useful for identifying a COVID-19 patient. The extracted temperature and cough level were used to determine the beginning level of COVID-19. Most patients do not have the initial symptoms but are affected by COVID-19. Therefore, the remaining features are also required to identify the patient severity level. The derived features are the processed feature-selection approach to select the most relevant parts. In this stage, feature relevancy and correlation between the elements are examined to identify the appropriate feature. The feature-selection process minimizes the overfitting issues. Here, a predefined threshold value is used to determine the quality and helps to rank the features. The correlation between the features is estimated by using Equation (8).

(8) $$\mathrm{cc}=\frac{(\mathrm{NF}\left(\sum X_{i}{\times X}_{i+1}))-(\sum X_{i})\left(X_{i+1}\right)\right)}{\sqrt{\left[\mathrm{NF}\left(\sum X_{i}^{2}\right)-\left(\sum X_{i}^{2})]\times [\mathrm{NS}(\sum X_{i+1}^{2}\right)-\left(\sum X_{i+1}^{2}\right)\right]}},i=1,\;2,\;\mathrm{NF}$$

The correlation between the features is computed from the total number of features (NF) and the position of the feature variable (X_i_). After computing the feature correlation, it has to be compared with the threshold value to rank the feature. The ranking process (Fit Rate (*FR*)) is performed using Equation (9).

(9) $$FR=\left(Sort\;\left(cc\right)\right)\;>threshold$$

The derived features are fed into the meta-heuristic optimized neural model to classify the COVID-19 patient health condition.

### 3.3. Health-Status Classification

**Figure 3:**
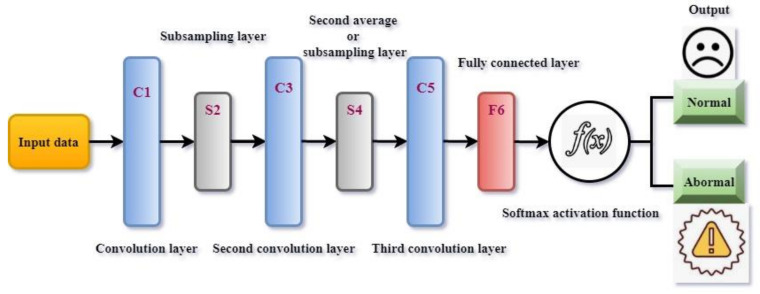
Process of convolute neural network.

Here, a salp optimized convolute neural network is utilized to investigate the selected features. The convolute neural model is a deep-learning technique used to perform the classification task. The convolute network effectively processes the raw input without requiring any processed information. The network automatically extracts the features from the input and previously trained details. These are the main reasons for selecting the convolute network to analyze the wearable sensor information. This network is a feed-forward network; hence, it utilizes a minimum of 20 to a maximum of 30 layers. The network primarily consists of convolution layers arranged on top of each other. This convolution layer helps to identify the sophisticated and complex information effectively. According to the discussion, the convolute network output is illustrated in Figure 3.

As Figure 3 shows, the convolute network consists of several layers that help to process the incoming health input. The first layer is C1, the convolution layer with six 5 × 5 size kernels. These convolution layers help to identify the normal features from the input data. This layer is the key building of the network. The convolute layer investigates the patterns from the extracted features. The derived features are mapped with the kernel patterns to identify the patient health status. If the kernel gives a larger positive value, no matching pattern is presented; otherwise, the kernel gives 0 or minimum value. The extracted value is fed into the second layer, S2, i.e., the subsampling layer. This layer is named the average pooling layer; this step minimizes the number of inputs by performing the average process. The minimized inputs are fed into the second convolution layer, C3. This layer has 16 convolution layers with a 5 × 5 size used to investigate the more relevant characteristics of the features.

Further, the derived features are fed into the second average pooling layer, S4. The S4 layer scales down the features and minimizes the number of features. Then C5 and F6 fully connected layers with 120 nodes are utilized to identify the nearer output for the given input. Finally, the identified output is processed by the SoftMax activation function defined in Equation (10) to find the exact output.

(10) $$S_{x}=\frac{1}{1+e^{-x}}$$

The identified output *S*(*x*) is the effect of the non-linearity adding in the convolute network. The activation function helps to avoid the condensed down of the network. The computed output is evaluated in the maximum function to determine the net output value of the given input. If the maximum value returns, the patient is affected by COVID-19; otherwise, he/she is normal. Once the patient is affected by COVID-19, the patients and relatives are alerted via SMS/email. The computed output is compared with the trained data during the output estimation process to minimize the deviation. If the system has variation, that is reduced by updating the neural parameters, such as weight and bias value. Here, the salp optimization algorithm is utilized to update the network parameter. The optimization technique converges the convolute network functionality and maximizes the disease recognition speed by making the feature learning. The algorithm works according to the behavior of the salp fish-food searching process. The salp food-hunting process helps to perform the network parameter updating process. Let *F* = {*F*_1_, *F*_2_, …, *F_n_*} be the number of COVID-19 patients features reserved for feature learning. The *F* is treated as a salp fish population.

(11a) $$F_{j}=Fit_{j}+c_{1}\left(F_{max}-F_{min}\right)\times c_{2}+F_{min}\;\;\;\;\;\;\;c_{3}\geq 0\;$$

(11b) $$F_{j}=Fit_{j}-c_{1}\left(F_{max}-F_{min}\right)\times c_{2}+F_{\min\;}\;\;\;\;\;\;c_{3}<0$$

In the food-searching process, the fish position is updated according to Equation (11a) when the coefficient ($c_{3}\geq 0),\;\mathrm{while}\;\mathrm{we}\;\mathrm{use}\;\mathrm{equation}\;\left(11b\right)\;\mathrm{if}\;\mathrm{the}\;\mathrm{coffecient}\;(c_{3}<0),$ this process is used to update the convolute network weight value. $F_{j}$ is defined as the best-updated weight value, which is computed with the help of the fitness solution [Fit]_j_, minimum $F_{min}$, and maximum $F_{max}$ COVID-19 patient feature value. The network function is regularized during this process, using random coefficients such as $c_{1}$, $c_{2}$, and $c_{3}$. These coefficients decrease or increase the network weight value, ranging from 0 to 1. The third coefficient, $c_{3},$ helps determine the weight-updating criteria among the three coefficients. If the $c_{3}$ value is greater than 0, then it has to be updated according to equations (11a, 11b) is used to update the weight value. Here, the coefficient $c_{1}$ is derived as in Equation (12). The initial network weight value is estimated using the current and maximum iteration during the feature learning.

(12) $$c_{1}=2e^{-{\left(\frac{4\times current\;iteration}{Maximun\;iteration}\right)}^{2}\;\;\;\;\;\;\;\;\;\;\;\;\;\;\;\;\;\;\;\;\;\;\;\;\;\;\;\;\;}$$

Then the remaining nodes’ weight value is computed using Equation (13).

(13) $${\left(F_{j}\right)}^{i}=\frac{1}{2}\times {\left(at\right)}^{2}+t\times v_{0},\;i\geq 2,\;{\left(F_{j}\right)}^{i}$$

The remaining nodes’ weight values are defined from time (T), initial speed (V), and acceleration (a) variables. Here, “*i*” is represented as the number of COVID-19 patient features of n batch size. Weight-value-related acceleration and speed are computed as Equation (14).

(14) $$a=\frac{V_{end}}{V_{0}},\;\mathrm{and}\;V=\frac{D-D_{0}}{T}$$

In this model, the initial and the end speed limit are defined as $V_{0}$ and $V_{end}$. The variables ($F_{0})$ and (*F*) attain the starting and ending point to compute node weight as Equation (15).

(15) $${\left(F_{j}\right)}^{i}=\frac{1}{2}({\left(F_{j}\right)}^{i}+{\left(F_{j}\right)}^{i-1})$$

According to these salp processes, the network weight values are updated continuously. This process helps to minimize the deviation between actual and predicted output values.

(16) $$T-I\left(x,l\right)=T\left(x,l\right)\times \frac{I\left(x\right)}{N}$$

The detail of network weight values from database T are gathered, and then COVID-19 features (I) are gathered together. The database is then divided into two sets (x and I): one for training x and the other for testing I. A machine learning algorithm is used to process the training data N. The validated dataset is then classified and the final outcome is displayed in Equation (16).

(17) $$x=\beta +\overset{q^{p}}{\underset{p=0}{\sum }}g^{p}$$

(18) $$y=\beta +\overset{q^{p}}{\underset{p=-1}{\sum }}g^{p}$$

The collection of features is represented by the feature counts vectors *x* and *y* for the learning sample, where the initial state is specified as β. This model has been shown to be quick and effective for many text categorization issues with the process of applying summation with limits $p=-1\;to\;q^{p}$ and $p=0\;to\;q^{p},$ using Equations (17) and (18). It used a primary neural network with two encoding levels and a sigmoid transfer layer in this application $g^{p}$.

(19) $$h_{x}=\left(1-z_{x}\right)\times h_{x-1}+\left[z_{x}\times {\hat{h}}_{x}\right]$$

(20) $${\hat{h}}_{x}=\tanh\left[B_{{\hat{h}}_{x}}\left(h_{x-1}\times r_{x}\right)+V_{{\hat{h}}_{x}}\times p_{x}\right]$$

The hidden layer function is denoted as $h_{x}$. Two concealed layers, ${\hat{h}}_{x}$ and ${\hat{h}}_{x-1}$, are integrated into the weight values to retrieve the upcoming and previous contexts. The output layer feature is denoted as $z_{x}$. That allows for the flow of temporal data in both ways mentioned in Equations (19) and (20). The feature vector is depicted as $V_{{\hat{h}}_{x}}$.

The effective utilization of convolution and subsampling layers is more helpful in predicting the COVID-19 patient health condition with a minimum error rate. Then the discussed system effectiveness is evaluated by using experimental results and discussion.

## 4. Results and Discussion

This system uses different sensors for collecting self-isolated patient health details. The data collection is carried out by a three-layer IoT designed framework illustrated in Figure 2. The gathered information is transmitted via API and Internet connection. The detailed IoT-based data-collection process is illustrated in Figure 1. This health information was further investigated using a meta-heuristics optimized convolute neural network (MHCNN). The analyzed system was developed using a MATLAB implementation tool with COVID-19 Open Research Dataset [31]. The dataset information helps train the neural model to analyze and classify the real-time patient health details. The dataset examines 4700 scholarly articles to gather patient health information. The global research community listed the COVID-19 patient health condition, which was used to learn the neural network. From the collected data, 30% of information was used for training, and 70% was used as a testing dataset. The system’s excellence is evaluated using various performance metrics defined in the following equations (21)-(23). In this research, preprocessing tools were used for computing numerical analysis based on runtimes and computational aspects.

(21) $$Mean\;square\;error\;\left(MSE\right)=\frac{\sum_{i=1}^{n{\left(Actual\;class{\;}_{i}-Predicted\;\;class{\;}_{i}\right)}^{2}}x}{no.of\;class}$$

(22) $$\mathrm{FR}=\frac{1-RMSE}{\sqrt{\frac{1}{(number\;of\;samples\sum \left(actual\;class-predicted\_mean\;\right)}}}\times 100\%\;$$

Were

(23) $$Correctly\;predicted\;True\;class\;\left(CPTC\right)\;Wrongly\;predicted\;True\;class\;\left(WPTC\right)\;Correctly\;predicted\;False\;class\;\left(CPFC\right)\;Wrongly\;predicted\;False\;class\;\left(WPFC\right)\;Accuracy=\left[\frac{(CPTC+WPTC)}{\left(CPTC+WPTC+WPFC+WPFC\;\right)}\right]$$

By using the above performance metric, the introduced meta-heuristics optimized convolute neural network (MHCNN) approach is compared with research approaches used in previous studies, such as the end-to-end deep-learning model (ETEDL) [20], B5G and explainable deep-learning model (BG5D EDL) [21], and optimized deep Bayes squeeze network (ODBSN) [25].

**Figure 4:**
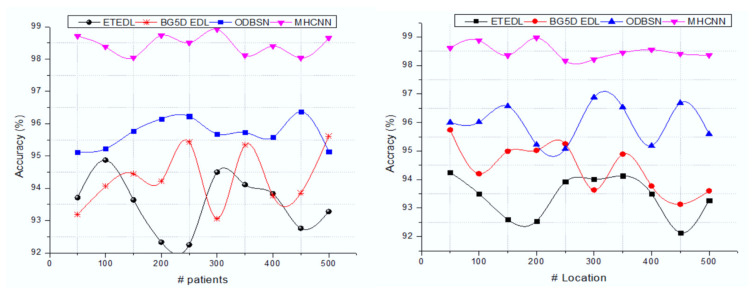
Accuracy analysis with various numbers of patients and locations.

Figure 4 illustrates the accuracy analysis of the IoT-based remote patient-health-monitoring system of the MHCNN approach. Here the comparison is made on a different number of patients and various locations. The created system achieves better results on both conditions (patient and location). This process extracts the MFCC features from an audio signal that helps to investigate the cough level. The MFCC features are varied from a normal, dry, and severe cough, and respective optimized features are selected according to the number of features and the respective ratio of features. In addition to this, ranked features improve the overall classification accuracy (98.72%). Furthermore, Table 2 examines overall system effectiveness.

Table 2 illustrates that the introduced system ensures a minimum error rate because the convoluted network uses the effective training model and optimized weight and bias updating process. The network-parameter weight values are updated according to Equation (24)

(24) $${\left(D_{j}\right)}^{i}\;=\;\frac{1}{2}\;{\left(at\right)}^{2}+t\times v_{0},\;i\geq 2,{\left(D_{j}\right)}^{i}$$

The ideal rule of thumb is one decimal place and two significant digits. When comparing group averages or percentages in tables, rounding should not obscure their differences. The salp optimization techniques help identify exact network parameters according to the random coefficient, which helps to improve the actual class. It is true that in a two-by-two situation, they may be reduced to the phi coefficient, but this is a one-way street. Between −1 and +1, the Matthews correlation coefficient shows complete agreement or disagreement, while between 0 and 1, it indicates no connection. The distribution of the two variables determines the maximum value of the coefficient if one or both variables may take on more than two values.

**Figure 5:**
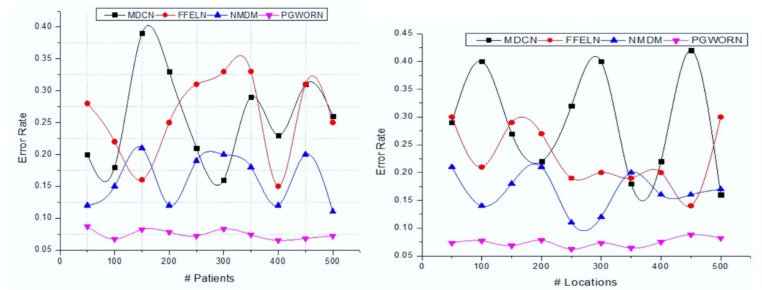
Error-rate analysis with a various number of patients and locations.

Then the deviation between actual and predicted values is evaluated using error values, and Figure 5 illustrates the respective graphical analysis.

Figure 5 illustrates the error-rate analysis of the IoT-based remote patient health monitoring system of the MHCNN approach. The comparison was performed on the number of patients and various location-related patients. This system produces the minimum error values while analyzing IoT-related health datasets, which exhibit a consistent up-and-down pattern as the sine (or derivative) function changes. An entire sinusoidal waveform is formed by projecting the various rotational positions of 0° and 360° to the waveform’s vertical axis when the wire loop or coil completes a full revolution or 360 degrees of rotation. The successful convolute learning model and training process predict the actual output for incoming inputs. The minimum error value indicates that the system recognizes the patient’s health condition with the highest accuracy measure. The system’s effectiveness was evaluated using the fit rate (FR). Table 3 illustrates the results obtained for the various patients.

**Figure 6:**
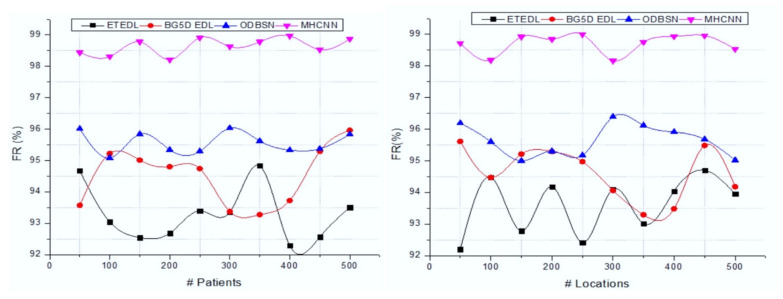
Fit-rate analysis with various numbers of patients and locations.

Table 3 clearly shows that the introduced MHCNN approach attains high FR-rate (98.7%) accuracy on various patients. The MHCNN approach utilizes almost 500 patients’ health information using the wearable sensor. The collected details are investigated using the MFCC approach, which derives the features using predefined filters and coefficient characteristics. The extracted features are examined by using the feature-rank process. This process computes the correlation between features that help to identify COVID-19-relevant fit features. As observed from Table 1 and Table 2, all the data were validated using preprocessing software. If the original data are mixed, round to one decimal place more than the least precise. However, it is usually suitable for datasets of size roughly 10– > 30. Further, it helps to increase the degree of absolute precision. The number of digits has nothing to do with the “reliability” of any reported results; it has everything to do with conveying the result’s precision. Reporting the results with more digits than the original measurements implies greater precision. The MHCNN approach uses the salp optimization technique that helps minimize the convergence issues in the neural model. Figure 6 illustrates the respective graphical analysis of the fit rate.

Figure 6 demonstrates the fit-rate comparison curve of various methods, such as the end-to-end deep-learning model (ETEDL) [20], B5G with an explainable deep-learning model (BG5D EDL) [21], optimized deep Bayes squeeze network (ODBSN) [25], and the proposed MHCNN approach. This causes the minimization of the deviation error and improves the system’s overall accuracy. The effective utilization of full convolution and subsampling layers fit the features into respective classes with a more fit rate (98.76%). Thus, the introduced IoT wearable sensor-based remote health-monitoring system recognizes a COVID-19 patient with maximum accuracy and minimum error rate. In this research, MFCC-based derived features are utilized as an input to the neural network to recognize the patient’s health condition. Based on such an observation, 256 FFT points, 101 sliding widow size, 300 Hz MEL filter lowest band edge, 800 Hz MEL highest band edge, 0.98 pre-emphasizing coefficients, 1 shift, and 800 Hz sampling frequency/2 were utilized to derive the variation of the characteristics in the patient audio signal. Further in this research, the periodic Fourier function was utilized, as it helps to clip and digitize the content for effective characterization of feature changes over time, providing the context information. Moreover, a chopped frame with windowing techniques maintains the original frequency information better with less noise; therefore, it is represented by the sum of sinusoidal waves.

## 5. Conclusions

The system uses the meta-heuristics optimized convolute neural network (MHCNN) approach to classify the COVID-19-patient health condition. This work uses the three-layer IoT framework to gather patient health information. The wearable sensors collect patient physiological factors, such as temperature, heart rate, oxygen saturation, and audio signals. MFCC coefficients processed the gathered audio signal to derive the cough-level infections. Further statistical features are extracted to identify the various health details. Then the temperature and cough threshold values are used to investigate the particular COVID-19 infection features. Then the fully convolution layer, the subsampling, and the activation function are utilized to categorize the normal and abnormal health features. Salp optimization behavior is applied to update the neural network parameters during the classification process. The system developed by using the MATLAB tool ensures 98.76% accuracy with a minimum deviation rate. In the future, the COVID-19-disease recognition process will be improved by applying optimized learning and process to minimize the convergence and maximize overall accuracy.

### Limitations and Future Research

The COVID-19-disorder recognition system will be improved in the future by implementing optimized knowledge acquisition and processes to minimize integration and maximize accuracy results. This study shows that mindset and emotion recognition based on natural-language processing cannot only reveal potential cross-cultural patterns. Social media platforms can also link consumers’ sentiments to actual events with high certainty. During a global recession, such as the coronavirus flu epidemic, there is a clear relationship between the opinions expressed despite social and political distinctions. Then the temperature and cough threshold values are used to investigate particular COVID-19 infection features, which is considered a major limitation of this proposed work.

## Figures and Tables

**Table 1:** Comparison analysis on conventional methods.

Method	Advantages	Disadvantages
ETEDL	Integrates the fog, cloud, and wireless body network and uses clinical decision-making concepts.	This method can only track a patient’s health condition up to 97.5% accuracy.
BG5D EDL	Effective deep-learning architecture to support data privacy.	Three-phase reconciliation global DL framework that is less than our proposed work.
ODBSN	Reduce the false recognition rate and minimize the computation time.	A Bayesian optimization algorithm is utilized only during the classification process.
MHCNN	The effectiveness of an IoT wearable-sensor-based remote health-monitoring system for COVID-19 patient health conditions can be measured.	The temperature and cough threshold values are used to investigate particular COVID-19 infection features. This limitation is considered a major drawback of this proposed work.

**Table 2:** Overall efficiency.

Measure	Value	Derivations
Sensitivity	0.9998	TPR =TP/(TP + FN)
Specificity	0.9984	SPC = TN/(FP + TN)
Precision	0.9984	PPV = TP/(TP + FP)
Negative Predictive Value	0.9998	NPV = TN/(TN + FN)
False Positive Rate	0.0016	FPR = FP/(FP + TN)
False Discovery Rate	0.0016	FDR = FP/(FP +TP)
False Negative Rate	0.0002	FNR = FN/(FN + TP)
Accuracy	0.9991	ACC = (TP + TN)/(P + N)
F1 Score	0.9991	F1 = 2TP/(2TP + FP + FN)
Matthews Correlation Coefficient	0.9982	TPsTN − FP × FN/sqrt ((TP + FP) (TP + FN) (TN + FP) × (TN + FN))

**Table 3:** Effectiveness of the system.

Methods	Number of Patients									
	50	100	150	200	250	300	350	400	450	500
ETEDL	92.47	93.96	94.23	92.44	93.53	94.45	94.9	94.05	92.71	93.91
BG5D EDL	94.54	93.67	94.4	93.74	93.1	93.89	94.65	94.02	93.84	93.57
ODBSN	95.43	95.7	95.67	95.89	96.61	96.38	96.53	96.17	96.05	96.2
MHCNN	98.85	98.78	98.74	98.33	98.5	98.44	98.34	98.81	98.56	98.67
**Methods**	**Number of Locations**									
	**50**	**100**	**150**	**200**	**250**	**300**	**350**	**400**	**450**	**500**
ETEDL	94.28	94.6	94.25	94.83	93.53	92.61	94.65	94.93	94.58	93.37
BG5D EDL	93.55	94.8	94.12	95.75	94.96	94.02	93.87	93.71	93.96	95.4
ODBSN	96.75	95.7	96.33	96.46	96.1	96.98	95.39	95.91	96.73	96.51
MHCNN	98.35	98.78	98.67	98.31	98.74	98.32	98.27	98.48	98.44	98.3

## Data Availability

The data presented in this study are available upon request from the corresponding author.
